# Identification of Tmem10/Opalin as a novel marker for oligodendrocytes using gene expression profiling

**DOI:** 10.1186/1471-2202-9-40

**Published:** 2008-04-25

**Authors:** Angelika Kippert, Katarina Trajkovic, Dirk Fitzner, Lennart Opitz, Mikael Simons

**Affiliations:** 1 Centre for Biochemistry and Molecular Cell Biology, University of Göttingen Humboldtallee 23, Göttingen, Germany; 2 Max-Planck-Institute for Experimental Medicine, Hermann-Rein-Str. 3, Göttingen, Germany; 3 Department of Neurology, University of Göttingen, Robert-Koch-Str. 40, Göttingen, Germany

## Abstract

**Background:**

During the development of the central nervous system, oligodendrocytes generate large amounts of myelin, a multilayered insulating membrane that ensheathes axons, thereby allowing the fast conduction of the action potential and maintaining axonal integrity. Differentiation of oligodendrocytes to myelin-forming cells requires the downregulation of RhoA GTPase activity.

**Results:**

To gain insights into the molecular mechanisms of oligodendrocyte differentiation, we performed microarray expression profiling of the oligodendroglial cell line, Oli-neu, treated with the Rho kinase (ROCK) inhibitor, Y-27632 or with conditioned neuronal medium. This resulted in the identification of the transmembrane protein 10 (Tmem10/Opalin), a novel type I transmembrane protein enriched in differentiating oligodendrocytes. In primary cultures, Tmem10 was abundantly expressed in O4-positive oligodendrocytes, but not in oligodendroglial precursor cells, astrocytes, microglia or neurons. In mature oligodendrocytes Tmem10 was enriched in the rims and processes of the cells and was only found to a lesser extent in the membrane sheets.

**Conclusion:**

Together, our results demonstrate that Tmem10 is a novel marker for in vitro generated oligodendrocytes.

## Background

Oligodendrocytes are specialized cells of the central nervous system that produce myelin, a multilayered membrane spirally ensheathing axons and facilitating rapid nerve conduction [1,2]. The development of oligodendrocytes is a gradual process, in which each step of the differentiation process is characterized by stage specific markers [3-6]. Oligodendrocytes originate from oligodendrocyte precursor cells that arise from multiple foci along the neuronal tube and migrate into the future white matter of the brain. After reaching their final position, they develop into mature post-mitotic cells that produce the myelin sheaths [7]. During the progression through the oligodendroglial lineage, the cells loose their migratory and proliferative capacities and undergo dramatic changes in their morphology by the formation of a highly branched network of processes. This transformation is accompanied by the expression of a number of gene products that are highly enriched or even specific to oligodendrocytes such as the myelin basic protein (MBP), proteolipid proteins (PLP/DM20), myelin-associated glycoprotein (MAG), cyclic nucleotide phosphodiesterase (CNP) and the glycolipids, galactosylceramide and sulfatide. The capacity of oligodendrocyte precursor cells to differentiate into oligodendrocytes that express these different gene products is intrinsic to the lineage and occurs even in the absence of neurons [4,8]. Oligodendrocytes need to provide specific sorting and transport mechanisms to enable the synthesis of an extensive amount of myelin membrane in a very short time [3,9]. Since oligodendrocytes must produce myelin at the appropriate time of neuronal development, a number of reciprocal signalling systems are likely to operate to coordinate the organisation of axonal domains and the biogenesis of myelin [10-15]. A number of recent studies have shown that neuronal-derived signalling molecules control the development of myelin-forming glial cells [16-21]. We have recently shown that neurons regulate membrane trafficking in oligodendrocytes [19]. In the absence of neurons, the major myelin protein, PLP, is internalized and stored in late endosomes. After receiving an unknown soluble signal from neurons, oligodendrocytes reduce the rate of endocytosis and increase the retrograde transport of PLP from late endosomes to the plasma membrane. A fraction of PLP is released in association with exosomes [22,23]. Our previous work shows that changes in Rho GTPase activity were responsible for switching between these two modes of transport [24]. Inactivation of Rho GTPase activity reduced the transport of cargo to late endosomes and at the same time increased the mobilization of membrane from late endosomes. We found that a neuronal soluble factor was responsible for the downregulation of RhoA GTPase activity in the oligodendroglial cell line, Oli-neu [24]. The downregulation of RhoA function during morphological differentiation of oligodendrocytes is supported by a number of other studies [18,25]. In this study, we were interested in the transcriptional changes that occur after differentiation of Oli-neu cells by conditioned neuronal medium or by inactivation of Rho GTPase function. This effort led to the identification of the transmembrane protein 10 (Tmem10/Opalin) as a novel marker for oligodendrocytes. The transmembrane protein 10 is known as Tmem10/TMEM10 in mice, rats and humans, with the synonyms TMP10 or HTMP10. Recently four Tmem10 homologs of prosimian species (*Eulemur macaco*, *Lemur catta*, *Microcebus murinus *and *Otolemur garnetti*) have been named Opalin [26]. In this work the human, rat and mouse transmembrane protein 10 will be referred to as Tmem10.

## Results and Discussion

As a cellular model for oligodendrocyte differentiation we use the oligodendroglial cell line, Oli-neu. The advantage of this system is that morphological differentiation of a pure oligodendroglial culture can be triggered synchronously by adding conditioned medium from primary neuronal cultures to the cells. To characterize the gene changes that occur after incubation of Oli-neu cells with conditioned neuronal medium, we used Affymetrix microarrays. Oli-neu cells were incubated for 16 hours with conditioned neuronal medium and compared to untreated parallel cultures. Cell fractions were used to purify mRNA for microarray analysis (the data is available at NCBI Geo accession number GSE10291). Using a 1.7 fold cut-off, we found that 716 genes were up-regulated, whereas 386 genes were down-regulated by conditioned neuronal medium as compared to the control. The eighty most highly induced genes are shown in Table 1. We have previously shown that incubation of Oli-neu cells with conditioned neuronal medium leads to RhoA GTPase inactivation. We, therefore, performed gene expression profiling analysis after treating cells with the Rho kinase (ROCK) inhibitor, Y27632 for 16 hours and compared the transcriptional changes to the ones obtained after incubation with conditioned neuronal medium. Interestingly, 70% of the genes that were controlled by both conditioned neuronal medium and by treatment with Y27632 were regulated into the same direction, indicating that these treatments affected the fate of the cells into a common path (Table 2). Some of these genes such as the UDP galactosyltransferase 8a and CNP are known to be upregulated during the progression through the oligodendroglial lineage [4], whereas known housekeeping genes such as the ATP synthase served as internal controls and were not differentially expressed after incubation with conditioned neuronal medium or Y27632 (data not shown). One so far not characterized, but highly upregulated gene is the brain-specifically expressed, Tmem10 [27]. For our further study, we decided to focus on Tmem10 for a number of reasons. Tmem10 was the strongest up-regulated gene in our analysis of transcriptional changes induced by conditioned neuronal medium and as well highly up-regulated after treatment with Y27632. The analysis of the average fold up-regulation in both conditions shows that Tmem10 is the most up-regulated gene together with Cyp2c39 (cytochrome P450, family2, polypeptide 39) (Table 3). In addition, a previous microarray analysis identified a 23.75 fold up-regulation of Tmem10 during oligodendrocyte differentiation [28] and *in situ *expression data of Tmem10 in the Allen Brain Atlas  suggested enrichment in the white matter of the brain.

**Table 1:** Top 80 upregulated genes after addition of conditioned neuronal medium to Oli-neu cells

Probe set ID	Fold change	p-value	Gene name	Gene symbol
A_52_P624415	7.12	0.00015	transmembrane protein 10	Tmem10
A_51_P225761	6.96	0.00020	ESTs, no homologies found	
A_52_P225856	6.76	0.00013	ESTs, no homologies found	
A_52_P329250	6.51	0.00000	chromodomain helicase DNA binding protein 1	Chd1
A_51_P304109	6.41	0.00007	cytochrome P450, family 2, subfamily c, polypeptide 39	Cyp2c39
A_52_P160518	6.23	0.00005	Scm-like with four mbt domains 1	Sfmbt1
A_52_P771513	6.22	0.00027	ESTs, no homologies found	
A_52_P61864	6.14	0.00023	wingless-related MMTV integration site 2	Wnt2
A_51_P370640	6.05	0.00013	zinc finger, CCHC domain containing 5	Zcchc5
A_51_P186092	6	0.00017	male sterility domain containing 2	Mlstd2
A_52_P24076	5.95	0.00026	myotubularin related protein 7	Mtmr7
A_52_P448870	5.9	0.00025	RAB26, member RAS oncogene family	Rab26
A_52_P193256	5.81	0.00032	DNA segment, Chr 10, Brigham & Women's Genetics 0791	D10Bwg0791e
A_52_P350750	5.81	0.00017	cholinergic receptor, nicotinic, alpha polypeptide 4	Chrna4
A_52_P391098	5.74	0.00022	cAMP responsive element modulator	Crem
A_51_P127035	5.72	0.00082	RIKEN cDNA 4432405B04 gene	4432405B04Rik
A_52_P600304	5.69	0.00013	RIKEN cDNA 1200007B05 gene	1200007B05Rik
A_51_P448632	5.68	0.00033	RIKEN cDNA C030022K24 gene	C030022K24Rik
A_52_P188593	5.68	0.00017	hypothetical gene supported by AK049058; BC025881	LOC433886
A_51_P359002	5.59	0.00028	ESTs, no homologies found	
A_52_P302587	5.55	0.00021	chimerin (chimaerin) 2	Chn2
A_51_P444502	5.44	0.00029	immunoglobulin kappa light chain variable region Vk23	LOC381783
A_51_P461404	5.42	0.00014	SWI/SNF related, actin dependent regulator of chromatin	Smarca1
A_52_P577329	5.37	0.00021	RIKEN cDNA A230069A22 gene	A230069A22Rik
A_51_P334449	5.37	0.00033	olfactory receptor 50	Olfr50
A_52_P354306	5.32	0.00013	peroxisome biogenesis factor 26	Pex26
A_51_P462978	5.31	0.00033	membrane protein, palmitoylated 2	Mpp2
A_52_P661972	5.3	0.00021	RIKEN cDNA 9230112E08 gene	9230112E08Rik
A_52_P1133703	5.29	0.00014	CD47 antigen (Rh-related antigen)	Cd47
A_51_P169617	5.28	0.00041	TAF3 RNA polymerase II, TATA box binding protein	Taf3
A_51_P339934	5.24	0.00022	neurofilament, light polypeptide	Nefl
A_51_P472113	5.23	0.00060	ESTs, no homologies found	
A_51_P262563	5.22	0.00031	ESTs, no homologies found	
A_51_P284486	5.2	0.00030	glutathione S-transferase, mu 2	Gstm2
A_51_P103706	5.17	0.00054	cytochrome P450, family 2, subfamily c, polypeptide 29	Cyp2c29
A_52_P223626	5.17	0.00011	oligodendrocyte transcription factor 2	Olig2
A_51_P392209	5.17	0.00019	zinc finger protein 482	Zfp482
A_51_P283499	5.17	0.00022	dopamine receptor 4	Drd4
A_52_P229052	5.17	0.00019	transmembrane prot. with EGF-like and two follistatin-like	Tmeff2
A_52_P337910	5.15	0.00015	RIKEN cDNA E130114P18 gene	E130114P18Rik
A_51_P129108	5.11	0.00019	activating transcription factor 6	Atf6
A_51_P393934	5.11	0.00028	CD82 antigen	Cd82
A_51_P413005	5.08	0.00021	chimerin (chimaerin) 2	Chn2
A_51_P394574	5.05	0.00019	ESTs, no homologies found	
A_51_P478003	5.04	0.00046	poly(A) polymerase gamma	Papolg
A_52_P516733	5.02	0.00086	DNA segment, Chr 15, ERATO Doi 621, expressed	D15Ertd621e
A_52_P127776	5.01	0.00030	ESTs, no homologies found	
A_52_P384479	4.95	0.00014	leucine rich repeat and fibronectin type III domain	Lrfn5
A_52_P118323	4.93	0.00042	ESTs, no homologies found	
A_52_P685963	4.91	0.00041	tenascin R	Tnr
A_52_P313068	4.83	0.00016	RIKEN cDNA 8030462N17 gene	8030462N17Rik
A_51_P489107	4.83	0.00013	pleckstrin homology domain-containing, family A, memb. 2	Plekha2
A_51_P454008	4.82	0.00027	lipopolysaccharide binding protein	Lbp
A_52_P418956	4.81	0.00059	RIKEN cDNA 4933431E20 gene	4933431E20Rik
A_51_P244453	4.8	0.00059	potassium channel tetramerisation domain containing 3	Kctd3
A_51_P342206	4.79	0.00890	cytochrome P450, family 2, subfamily c, polypeptide 38	Cyp2c38
A_51_P270899	4.78	0.00019	zinc finger protein 61	Zfp61
A_52_P370162	4.78	0.00034	G protein-coupled receptor 23	Gpr23
A_52_P356170	4.77	0.00024	glyceraldehyde-3-phosphate dehydrogenase, spermatogenic	Gapdhs
A_51_P130254	4.75	0.00021	pleckstrin and Sec7 domain containing 3	Psd3
A_51_P363461	4.73	0.00022	ESTs, no homologies found	
A_52_P502838	4.72	0.00033	mannoside acetylglucosaminyltransferase 5	Mgat5
A_51_P494122	4.69	0.00050	RIKEN cDNA 1810009K13 gene	1810009K13Rik
A_52_P285194	4.69	0.00021	ESTs, no homologies found	
A_51_P169087	4.67	0.00035	ESTs, no homologies found	
A_51_P506822	4.65	0.00015	UDP galactosyltransferase 8A	Ugt8a
A_52_P164709	4.64	0.00030	WD repeat domain 51A	Wdr51a
A_51_P226269	4.62	0.00022	RIKEN cDNA 1190002H23 gene	1190002H23Rik
r60_a9	4.55	0.00003	fibronectin 1	Fn1
A_51_P111233	4.49	0.00013	dopamine receptor 2	Drd2
A_52_P265556	4.49	0.00042	predicted gene, ENSMUSG00000056850	ENSMUSG00000056850
A_52_P417654	4.47	0.00022	transcription elongation factor A (SII) 1	Tcea1
A_52_P625249	4.44	0.00021	cytochrome P450, family 2. subfamily c, polypeptide 37	Cyp2c37
A_52_P603038	4.44	0.00021	oligodendrocyte transcription factor 1	Olig1
A_51_P199199	4.42	0.00024	phosphoinositide-3-kinase adaptor protein 1	Pik3ap1
A_52_P510706	4.41	0.00030	DnaJ (Hsp40) homolog, subfamily A, member 2	Dnaja2
A_52_P57416	4.39	0.00044	ESTs, no homologies found	
A_52_P322639	4.38	0.00031	ESTs, no homologies found	
A_51_P232901	4.37	0.00007	cyclic nucleotide phosphodiesterase 1	Cnp1

**Table 2:** Genes regulated in the same direction after treatment with conditioned neuronal medium (cnm) or Y27632

	**Cnm**		**Y27632**			
Probe set ID	Fold change	p-val	Fold change	p-val	Gene name	Gene symbol
A_52_P624415	7,12	0,00015	2,2	0,00054	transmembrane protein 10	Tmem10
A_51_P304109	6,41	0,00007	2,96	0,00127	cytochrome P450, family 2, subfamily c, polypept. 39	Cyp2c39
A_52_P302587	5,55	0,00021	1,48	0,00111	chimerin (chimaerin) 2	Chn2
A_52_P229052	5,17	0,00019	2,05	0,00067	transmembr. prot. with EGF-like, dom. 2	Tmeff2
A_51_P103706	5,17	0,00054	1,29	0,00429	cytochrome P450, family 2, subfamily c, polypept. 29	Cyp2c29
A_51_P413005	5,08	0,00021	1,43	0,00086	chimerin (chimaerin) 2	Chn2
A_51_P454008	4,82	0,00027	1,37	0,00043	lipopolysaccharide binding protein	Lbp
A_51_P506822	4,65	0,00015	1,25	0,00296	UDP galactosyltransferase 8A	Ugt8a
A_51_P232901	4,37	0,00007	1,47	0,00096	cyclic nucleotide phosphodiesterase 1	Cnp1
A_52_P661327	4,31	0,00018	1,3	0,00029	phytanoyl-CoA hydroxylase interacting protein-like	Phyhipl
A_51_P433194	3,43	0,00024	1,46	0,00218	breast carcinoma amplified sequence 1	Bcas1
A_51_P437079	3,07	0,00013	1,44	0,00068	RIKEN cDNA 5730559C18 gene	5730559C18Rik
A_52_P269003	2,53	0,00062	1,45	0,00147	Neogenin	Neo1
A_51_P259975	2,48	0,00007	1,54	0,00051	aspartoacylase	Aspa
A_52_P493854	2,42	0,0002	2,18	0,00084	potassium channel tetramerisation domain	Kctd4
A_52_P493857	2,39	0,00023	2,31	0,00111	potassium channel tetramerisation domain	Kctd4
A_51_P354354	2,29	0,00032	2,12	0,00172	galactose-3-O-sulfotransferase 1	Gal3st1
A_51_P112308	2,25	0,00014	1,26	0,00057	RIKEN cDNA 1810011O10 gene	1810011O10Rik
A_51_P413721	2,18	0,00025	1,88	0,00036	gap junction membrane channel prot. epsilon 1	Gje1
A_51_P145376	2,12	0,00024	1,48	0,00033	OTU domain containing 7B	Otud7b
A_52_P168953	2,07	0,00037	1,38	0,0073	Versican	Vcan
A_52_P376169	2	0,00016	1,23	0,00125	LY6/PLAUR domain containing 6	Lypd6
A_51_P196596	1,95	0,00022	2,42	0,00046	tripartite motif protein 2	Trim2
A_51_P159453	1,94	0,00026	1,68	0,00202	serine (or cystein) peptidase inhib. 3n	Serpina3n
A_52_P149801	1,92	0,00044	1,9	0,0007	phosphodiesterase 4B, cAMP specific	Pde4b
A_52_P121502	1,87	0,00045	1,34	0,00159	plasma membrane proteolipid	Pllp
A_52_P465012	1,76	0,00081	2,75	0,00029	protein phosphatase 2, SU B (PR 52), beta isoform	Ppp2r2b
A_51_P512119	1,73	0,00067	1,31	0,00255	cDNA sequence AF067063	AF067063
						
A_52_P213932	-4,18	0,00021	-1,85	0,00255	metallopeptidase with thrombospondin type 1	Adamts1
A_51_P426754	-4,17	0,00203	-2,89	0,00059	annexin A5	Anxa5
A_52_P520495	-4,09	0,00022	-3,34	0,0005	vascular cell adhesion molecule 1	Vcam1
A_51_P115462	-3,73	0,00027	-2,57	0,00091	spermatogen. associat. glut. (E)-rich prot. 6, ps 1	Speer6-ps1
A_52_P433119	-3,55	0,00313	-2,14	0,00092	spermatogenesis associat. glut. (E)-rich prot. 2	Speer2
A_51_P183571	-3,31	0,00022	-1,64	0,0013	serine (or cysteine) peptidase inhib. 1	Serpine1
A_52_P62037	-3,18	0,00032	-1,47	0,00052	annexin A2	Anxa2
A_52_P63948	-3,13	0,0002	-2,44	0,00058	cDNA sequence BC048651	BC048651
A_52_P148703	-3,09	0,00013	-1,66	0,00059	fer-1-like 3, myoferlin (C. elegans)	Fer1l3
A_51_P131408	-2,99	0,00014	-1,33	0,00168	tumor necrosis factor receptor superfamily,12a	Tnfrsf12a
A_51_P282584	-2,96	0,0002	-1,39	0,00137	Olfactomedin-like 2B	Olfml2b
A_51_P165342	-2,87	0,00035	-1,84	0,00135	annexin A2	Anxa2
A_52_P518949	-2,84	0,00044	-2,3	0,00035	similar to spermatogen. associat. glut. (E)-rich prot. 2	LOC381612
A_52_P771912	-2,84	0,00019	-2,18	0,00091	lymphocyte antigen 6 complex, locus C	Ly6c
A_52_P360440	-2,81	0,00063	-1,39	0,00052	fer-1-like 3, myoferlin (C. elegans)	Fer1l3
A_51_P182116	-2,79	0,00021	-2,97	0,00033	Down syndrome critical region homolog 1 (human)	Dscr1
A_52_P628885	-2,77	0,00027	-2,25	0,00072	brain and acute leukemia, cytoplasmic	Baalc
A_52_P385546	-2,64	0,0002	-1,32	0,00158	C1q-like 3	C1ql3
A_52_P594584	-2,59	0,00179	-2,09	0,00208	spermatogenesis associat. glut. (E)-rich protein 2	Speer2
A_51_P228472	-2,48	0,00028	-1,94	0,00069	insulin-like growth factor binding protein 3	Igfbp3
A_52_P196732	-2,43	0,00039	-1,57	0,00151	NIMA-related expressed kinase 6	Nek6
A_51_P181595	-2,38	0,00024	-1,77	0,00058	spermatogen. associat. glut. (E)-rich prot. 1, ps 1	Speer1-ps1
A_51_P435023	-2,37	0,00007	-1,44	0,0003	Ras association (RalGDS/AF-6) domain family 1	Rassf1
A_52_P661565	-2,36	0,00034	-3,42	0,00068	chloride intracellular channel 4 (mitochondrial)	Clic4
A_52_P230938	-2,34	0,00082	-2,02	0,00771	lymphocyte antigen 6 complex, locus C	Ly6c
A_52_P93256	-2,25	0,00047	-1,49	0,00054	angiopoietin-like 2	Angptl2
A_51_P384968	-2,18	0,00046	-1,34	0,00158	nerve growth factor recept. (TNFR superfam., m16)	Ngfr
A_52_P427640	-2,14	0,00009	-1,84	0,00035	serine (or cysteine) proteinase inhib. 3m	Serpina3m
A_51_P351896	-2,08	0,00022	-1,29	0,00214	RIKEN cDNA 1110032E23 gene	1110032E23Rik
A_51_P427663	-2,08	0,00022	-1,61	0,00043	calponin 2	Cnn2
A_51_P248441	-2,07	0,00061	-1,23	0,00047	ubiquitin-conjugating enzyme E2G 2	Ube2g2
A_51_P344263	-2,01	0,00079	-1,47	0,00115	brain and acute leukemia, cytoplasmic	Baalc
A_51_P411253	-2	0,00014	-1,73	0,00062	phosphoprotein enriched in astrocytes 15A	Pea15a
A_52_P201206	-2	0,00022	-1,31	0,00102	secernin 1	Scrn1
A_51_P517843	-1,94	0,00697	-1,43	0,00125	GLI pathogenesis-related 2	Glipr2
A_51_P503162	-1,9	0,00064	-1,48	0,00099	Kruppel-like factor 6	Klf6
A_52_P359088	-1,9	0,00704	-1,4	0,00314	solute carrier family 25 (mitochond., phosphat.)	Slc25a25
A_52_P617327	-1,76	0,00042	-1,54	0,00049	Down syndrome critical region homolog 1 (human)	Dscr1
A_52_P403157	-1,73	0,00131	-4,01	0,00029	sorbin and SH3 domain containing 2	Sorbs2
A_52_P246698	-1,72	0,00036	-1,6	0,00091	down-regulated by Ctnnb1, a	Drctnnb1a
A_51_P103819	-1,71	0,00173	-1,2	0,00036	similar to Tribbles homolog 2 (predicted)	RGD1564451_pred.

**Table 3:** Top 10 up-regulated genes co-regulated after treatment with conditioned neuronal medium (cnm) or ROCK inhibitor (Y27632)

	**cnm**	**Y27632**	**(cnm+Y27632)/2**		
Probe set ID	Fold change	Fold change	Avgerage Fold-change	Gene name	Gene symbol
A_52_P624415	7,12	2,2	**4,7**	transmembrane protein 10	Tmem10
A_51_P304109	6,41	2,96	**4,7**	cytochrome P450, family 2, subfamily c, polypept. 39	Cyp2c39
A_52_P302587	5,55	1,48	**3,5**	chimerin (chimaerin) 2	Chn2
A_52_P229052	5,17	2,05	**3,6**	transmembr. prot. with EGF-like, dom. 2	Tmeff2
A_51_P103706	5,17	1,29	**3,2**	cytochrome P450, family 2, subfamily c, polypept. 29	Cyp2c29
A_51_P413005	5,08	1,43	**3,3**	chimerin (chimaerin) 2	Chn2
A_51_P454008	4,82	1,37	**3,1**	lipopolysaccharide binding protein	Lbp
A_51_P506822	4,65	1,25	**3,0**	UDP galactosyltransferase 8A	Ugt8a
A_51_P232901	4,37	1,47	**2,9**	cyclic nucleotide phosphodiesterase 1	Cnp1
A_52_P661327	4,31	1,3	**2,8**	phytanoyl-CoA hydroxylase interacting protein-like	Phyhipl

**Figure 1:**
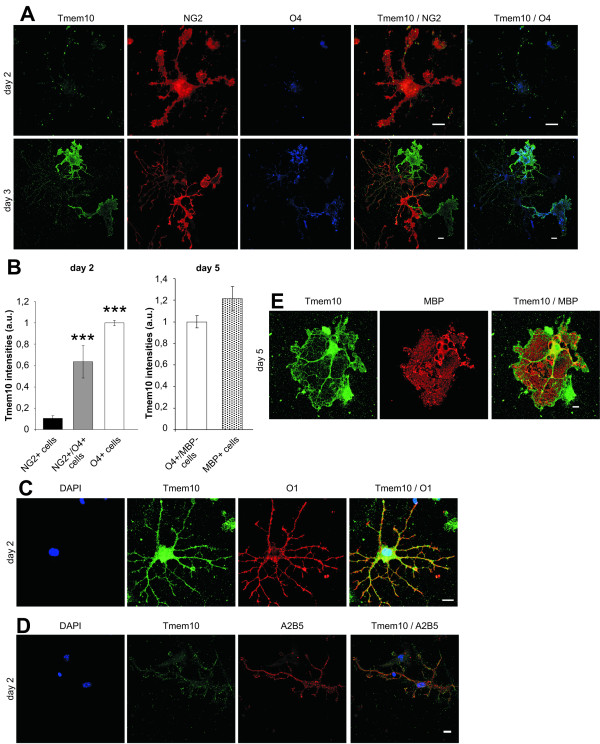
**Tmem10 is expressed during oligodendrocyte differentiation**. **(A) **Primary oligodendrocytes were cultured for 2–3 days and levels of Tmem10 (green) expression were determined by immunofluorescence. The developmental stage of the cells was determined by costaining for NG2 (red) or O4 (blue). **(B) **Quantitative analysis of the Tmem10 immunofluorescence intensities. Values represent the mean ± SEM (n > 20 cells, ***p < 0,001). **(C) **Tmem10 (green) is expressed in O1 (red) positive oligodendrocytes (day 2) and **(D) **Tmem10 (green) is weakly expressed in A2B5 (red) positive cells (day 2). **(E) **Tmem10 (green) localizes to processes and rims of mature oligodendrocytes, expressing MBP (red) (day 5). Scale bars, 10 μm.

To begin our characterization of Tmem10 we raised an antibody against the C-terminal part of the protein and performed immunofluorescence analysis on primary cultures of oligodendrocytes. We did not detect Tmem10 on NG2-positive oligodendrocyte precursor cells, whereas pre-oligodendrocytes that were still NG2-positive, but also contained O4 started to express Tmem10 (Fig 1A, B). Higher expression of Tmem10 was identified on NG2-negative and O4-positive or O1-positive oligodendrocytes (Fig 1A, C). Low levels of Tmem10 expression can be detected in A2B5-positive oligodendrocyte progenitors (Fig 1D). Colocalization studies of Tmem10 with MBP indicated that Tmem10 was present in mature oligodendrocytes where it was enriched in the rims and processes of the cells and was found only to a lesser extent in the membrane sheets (Fig. 1E). Comparisons of Tmem10 expression in O4-positive/MBP-negative and MBP-positive cells shows that Tmem10 is redistributed to the rims of the membrane sheets, but the expression level does not change significantly compared to O4-positive oligodendrocytes (Fig 1B).

**Figure 2:**
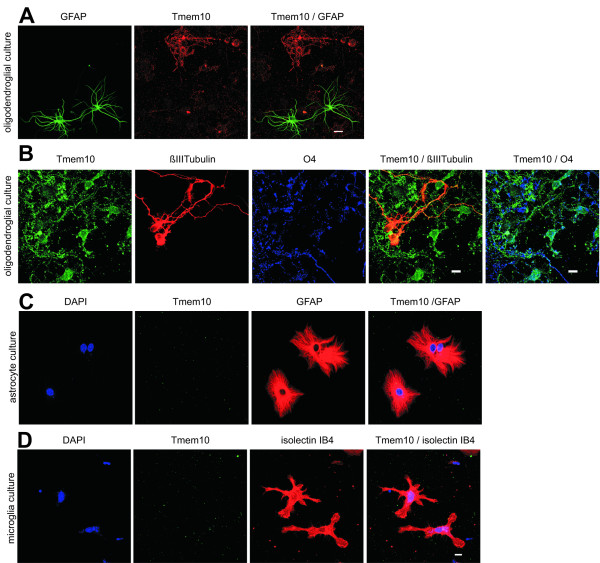
**Tmem10 is not detected on neurons, astrocytes and microglia**. **(A) **Tmem10 (red) is specifically expressed in oligodendrocytes, but absent form GFAP-positive astrocytes (GFAP, green). **(B) **Tmem10 (green) is enriched in oligodendrocytes, labelled with O4 (blue) compared to neurons stained for neuronal βIII Tubulin (red). Tmem10 (green) is not detectable on **(C) **astrocytes (GFAP, red) and **(D) **microglia (stained with isolectin IB_4 _conjugated to Alexa Fluor 568, red) in cultures enriched for these cells types. Scale bars, 10 μm.

Interestingly, double labelling of Tmem10 with GFAP or neuron specific βIII Tubulin showed that Tmem10 could not be detected on GFAP-positive astrocytes or neurons, which are present as a minor cell population in the same cultures, indicating a specific expression of Tmem10 in differentiating oligodendrocytes (Fig. 2A, B). Additionally, we analysed primary cultures of astrocytes and microglia cultures, but could not detect Tmem10 on either of these cell types (Fig. 2C, D).

**Figure 3:**
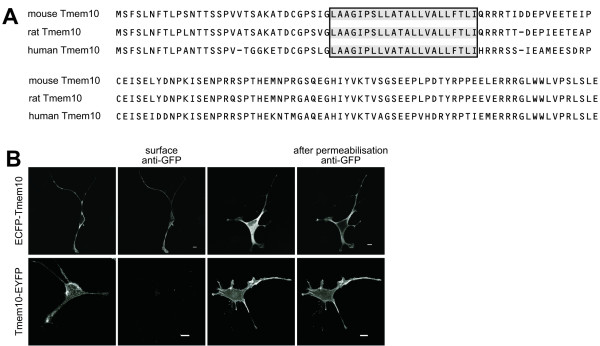
**Tmem10 is a type I transmembrane protein**. **(A) **Alignment of mouse, rat and human Tmem10 amino acid sequence. Black/grey box indicates the predicted position of the transmembrane domain. **(B) **Oli-neu cells were transfected to express either N-terminal ECFP-tagged or C-terminal EYFP-tagged Tmem10. Subsequent staining with anti-GFP antibody was performed either on living, unpermeabilized (surface anti-GFP) or on fixed, permeabilized (after permeabilization anti-GFP) cells. Scale bars, 10 μm.

The analysis of the primary amino acid sequence of Tmem10 for protein domains and functional sites with InterProScan [29] revealed a predicted signal peptide (amino acid 1–15 in the mouse sequence) and a putative transmembrane domain (amino acid 31–51 in the mouse sequence) (Fig. 3A). To further characterize the protein structure and the membrane orientation of Tmem10, we used N-terminal ECFP- and C-terminal EYFP-fusion proteins of Tmem10. Oli-neu cells were transfected with either of the fusion proteins and live staining was performed at 4°C with anti-GFP antibody to specifically label the proteins at the cell surface. We found that only the N-terminal ECFP-fusion protein was detectable by surface staining, whereas premeabilization of the cells uncovered both fusion proteins (Fig. 3B). These results show that Tmem10, as predicted from its primary amino acid sequence, is a type I membrane protein.

**Figure 4:**
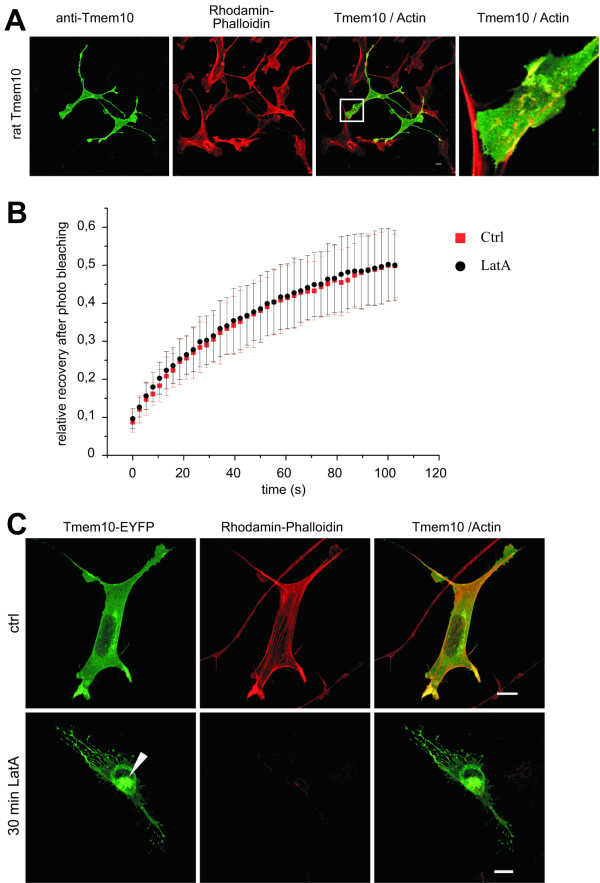
**Tmem10 colocalizes with actin in processes and membrane ruffles**. **(A) **Oli-neu cells were transfected with a plasmid encoding for rat Tmem10 and subsequently stained for Tmem10 (green) and Actin (red) with rhodamin-labelled phalloidin. Note the colocalization of Tmem10 with Actin in processes and membrane ruffles. The absence of Tmem10 labelling in untransfected cells shows the specificity of the generated anti-Tmem10 antiserum. **(B, C) **Oli-neu cells were transfected with Tmem10-EYFP and treated with 2 μM latrunculin A for 30 min 16 h after transfection. **(B) **FRAP was measured by bleaching a squared region of interest within the cell body and fluorescence recovery in this regions was examined. Average FRAP tracings for 15 cells form 2 independent experiments are shown. **(C) **Tmem10-EYFP (green) accumulates in intracellular sites (arrow head) after disruption of the F-actin (red) cytoskeleton with latrunculin A. Scale bar, 10 μm.

After ectopic expression of both Tmem10 fusion proteins and an untagged expression construct, we found that it was mainly localized at the plasma membrane with only very little intracellular staining. Tmem10 appeared to be enriched in actin-rich membrane ruffles at the cell surface as shown by its colocalization with rhodamine-phalloidin (Fig 4A). Additionally, comparison of Tmem10 transfected Oli-neu with untransfected control cells shows that our anti-Tmem10 antibody specifically recognizes Tmem10 (Fig 4A).

To investigate the role of the actin cytoskeleton on the lateral mobility of Tmem10 we performed fluorescence recovery after photobleaching (FRAP) experiments in the presence or absence of the F-actin disrupting drug, latrunculin A. The mobile fraction and the half time of fluorescence recovery did not change after treatment with latrunculin A (Fig 4B), indicating that the lateral mobility of Tmem10 within the plasma membrane is not dependent on a functional cortical actin cytoskeleton. Interestingly, we did observe a redistribution of a fraction of plasma membrane Tmem10 into intracellular sites after treatment with latrunculin A (Fig 4C), suggesting a role of the actin cytoskeleton in keeping Tmem10 at the cell surface.

In summary, we have applied an expression profiling approach to identify genes upregulated during Oli-neu cell differentiation. We used two different experimental approaches – incubation with conditioned neuronal medium or treatment with the Rho kinase (ROCK) inhibitor, Y-27632. This procedure led to the identification of Tmem10, a protein that appears during oligodendrocyte differentiation. In cultured oligodendrocytes, Tmem10 was absent from bipolar precursor cells and started to be expressed after the cells had acquired the O4 epitope.

A previous comparative genome analysis suggested that Tmem10 is a mammalian-specific gene [26]. Interestingly, the comparison of the genome structure of the Tmem10 gene and its flanking region identified an evolutionary conserved region within the first intron that functions as an oligodendrocyte-specific enhancer. This domain contains binding sites for Myt1 and cAMP-response element binding protein (CREB) and the treatment of Oli-neu cells with cAMP enhanced the expression of Tmem10 [26]. Previous studies have already shown that cAMP regulates the expression of several other oligodendroglial-specific genes [30], suggesting a general role for cAMP dependent signalling in the differentiation of oligodendrocytes into myelin-forming cells. Another factor that appears to regulate Tmem10 gene expression was the leukaemia inhibitory factor (LIF) [26], which seems to be released by astrocytes in response to ATP secreted by neurons and to promote myelination by mature oligodendrocytes [31]. Together, these data suggest a function for Tmem10 in an oligodendroglial specific process. Our finding that Tmem10 colocalizes with F-actin in plasma membrane ruffles and in F-actin-rich processes, points to a role in the regulation of the oligodendroglial actin cytoskeleton. This is reminiscent to another oligodendroglial-specific protein, Ermin, which has been implicated in the regulation of cell morphology by modulating the actin cytoskeleton [32]. The localization of Tmem10 to the leading edge of myelin sheets in mature oligodendrocytes suggests a role for Tmem10 in myelin membrane sheet extension. As Tmem10 localizes to the growing tip of the myelin sheet it could also be involved in the process of recognition or adhesion to potential axonal targets. Further analysis will be required to elucidate these issues.

## Conclusion

During the development of the nervous system oligodendrocytes form a highly branched network of processes and several oligodendroglial-specific genes such as Ermin, CNP and Tmem10 are expressed during this process. Gene profiling using microarrays is a useful starting point to identify genes relevant to oligodendrocyte differentiation and myelination [28,33,34]. Functional analysis of these proteins, as performed for CNP [35], will be required to elucidate their exact biological function in the generation of myelin-forming processes.

## Methods

### Cell culture, transfections and immunofluorescence

Primary cultures of mouse oligodendrocytes were prepared as described previously [36]. In brief, cells were plated in MEM containing B27 supplement, 1% horse serum, L-thyroxine, tri-iodo-thyronine, glucose, glutamine, gentamycine, pyruvate, and bicarbonate on poly-L-lysine coated glass-coverslips after shaking. The minor population of neurons and astrocytes which arise together with the oligodendrocytes in the mixed brain cultures were used to assess Tmem10 expression in other cell types. Primary cultures of microglia and astrocytes were prepared as described previously [37]. In brief, microglial cells were shaken off, centrifuged and plated on poly-L-lysine coated coverslips in D-MEM containing 10% FCS, glutamine, penicillin and streptomycin. The remaining astrocytes were trypsinized, centrifuged and plated on poly-L-lysine coated coverslips in D-MEM containing 10% FCS, glutamine, penicillin and streptomycin. The oligodendroglial cell line Oli-neu was cultured as described previously [38]. For microarray experiments we used Oli-neu cells stably expressing PLP-EGFP [19]. Cells were treated for 16 h with conditioned neuronal medium or Y27632 (Calbiochem). Conditioned neuronal medium was obtained from primary cultures enriched in neurons after culturing for 2 weeks and used directly as described previously [24]. Transient transfections were performed using FuGENE transfection reagent (Roche Diagnostics, Basel, Switzerland) according to the manufacturer's protocol. Immunofluorescence was performed as described previously [19]. For surface labelling of ECFP-Tmem10 or Tmem10-EYFP transfected, living cells were incubated with anti-GFP antibody in medium for 10 min at 4°C, washed, fixed and labelled with secondary antibody. Disruption of the actin cytoskeleton was done 16 h after transfection with 2 μM latrunculin A for 30 min at 37°C.

### Antibodies and plasmids

The following plasmids were used: human Tmem10 cDNA C-terminally fused with EYFP or N-terminally fused with ECFP [39] generated from modified pECFP-C1 or pEYFP-N1 expression vectors (Clonetech, Heidelberg, Germany), rat Tmem10 cDNA subcloned in pExpress-1 expression vector purchased form RZPD (Deutsches Ressourcenzentrum für Genomforschung GmbH, Berlin, Germany).

Anti-Tmem10 antiserum was induced in rabbits against the C-terminal Tmem10 sequence LERRRGLWWLVPSLSLE and the affinity purified IgG fraction was used. Peptid synthesis, immunization of the rabbit and affinity purification was carried out by Davids Biotechnology (Regensburg, Germany). Further the following primary antibodies were used: A2B5 (mouse monoclonal IgM, Chemicon (Millipore)), GFAP (mouse monoclonal IgG1; Vision BioSystems Novocastra, New Castle upon Tyne, UK), GFP (rabbit polyclonal IgG; Abcam, Cambridge, UK), MBP (mouse monoclonal IgG1; Sternberger Inc., Lutherville, MD), NG2 (rat IgG) [40], O1 (monoclonal IgM) [41], O4 (monoclonal IgM) [41], Tmem10 (rabbit polyclonal IgG fraction), neuron specific βIII Tubulin (mouse monoclonal IgG1; Promega, Madison, WI). Microglial cells were stained with isolectin IB_4 _conjugated to Alexa Fluor 568 from Molecular Probes (Invitrogen, Carlsbad, CA). Secondary antibodies were purchased from Dianova (Hamburg, Germany) and rhodamin-labelled phalloidin from Molecular Probes (Invitrogen, Carlsbad, CA).

### RNA isolation

RNA isolation was performed using the Trizol (Invitrogen, Carlsbad, CA) method according to the manufacturer's recommendations and stored at -80°C. Afterwards, the samples were DNAse I treated in order to remove genomic DNA contaminations. RNA quality was determined using the Agilent 2100 Bioanalyzer (Agilent Technologies) microfluidic electrophoresis. Only sample pairs with comparable RNA integrity numbers were selected for microarray analysis.

### Experimental design and sample preparation for 2 colour-microarrays

For gene expression profiling, a two-colour 1 × 2 design including a dye swap using 6 arrays was applied, comparing Oli-neu cells stably expressing PLP-EGFP treated for 16 h with conditioned neuronal medium or Y27632, respectively, to untreated parallel cultures.

The samples for hybridization were prepared from total RNA according to the Atlas SMART Fluorescent Probe Amplification Kit (Clonetech-Takara Bio Europe) protocol, except, that the RNA template was hydrolyzed under alkaline conditions before cDNA purification, and the PCR amplification process was monitored and stopped in the exponential phase. Quantity and Cy-dye incorporation rates of the generated target material were assessed using a NanoDrop ND-100. Cy3- and Cy5-labelled cDNA fragments, respectively, were hybridized to Agilent Technologies 44 K Mouse Whole Genome Microarrays (G4112A) for 17 h at 65°C. Post-processing washes were done according to the Agilent Technologies SSPE protocol (v2.1), replacing wash solution 3 by acetonitril, followed by immediate scanning using an Agilent G2505B scanner. Intensity data were extracted using the software 'Automatic Image processing for Microarrays'.

### Statistical analysis

Normalization of the raw intensity data was done with a non-linear loess regression [42].

### Uni- and multivariate designs

Differentially expressed genes were identified by an ANOVA fixed effects model [43]. The resulting P-values were adjusted with the Benjamini-Hochberg method to control the False-Discovery-Rate [44]. Normalization and statistical computation was done for two independent datasets derived from a high gain and a low gain scan, allowing replacement of saturated features in the high gain scan with data from the low gain measurement.

### Sequence analysis

The prediction of the transmembrane domain of Tmem10 was done using the InterProScan algorithm provided at EMBL-EBI [29].

### Microscopy and analysis

Fluorescence images were acquired on a confocal laser scanning microscope (TCS SP equipped with AOBS, Leica) with a 40× or 63× oil plan-apochromat objective (Leica). Image processing and analysis was performed using Meta Imaging Series 6.1 software (Universal Imaging Corporation). Quantification of fluorescence intensities was performed as described previously [19]. FRAP experiments were done as described in [20].

## Authors' contributions

AK carried out cell culture, transfections and immunofluorescence, including microscopy and statistical analysis, performed amino acid sequence alignment, created figures and participated in drafting the manuscript. KT performed cell culture, RNA isolation and sample preparation for gene expression profiling experiments. DF carried out FRAP experiments. LO participated in analysis and presentation of gene expression data. MS developed the design of the study, deduced interpretation of the data and wrote the manuscript.
